# Pheochromocytoma Associated With Neurofibromatosis Type 1: A Case Report

**DOI:** 10.7759/cureus.87050

**Published:** 2025-06-30

**Authors:** Anwarul Bari, Marjan Jamila, Anik Barua Deepta, Adiat Atkia Abida Chowdhury, Hironmoy Barman

**Affiliations:** 1 Medicine, Sir Salimullah Medical College, Dhaka, BGD

**Keywords:** adrenal mass, case report, endocrine disorder, genetic disorder, neurofibromatosis type-1, pheochromocytoma, rare complication

## Abstract

We report a complex case of pheochromocytoma in a 50-year-old woman with a longstanding diagnosis of neurofibromatosis type 1 (NF-1), characterized by multiple cutaneous neurofibromas and axillary freckling. NF-1 is an autosomal dominant disorder that infrequently presents with pheochromocytoma, a potentially life-threatening endocrine tumor. The patient’s clinical presentation included recurrent abdominal pain, unintentional weight loss, and poorly controlled hypertension despite longstanding antihypertensive treatment and management of type 2 diabetes mellitus. What makes this case unique is the size of the tumor (10 cm), the initial misinterpretation as a renal mass, and the delay in diagnosis despite classical symptoms. Diagnostic workup revealed a palpable right renal mass, with ultrasound and computed tomography confirming a large adrenal mass. Elevated urinary metanephrines established the diagnosis of pheochromocytoma. This case underscores the necessity for clinicians to maintain a high index of suspicion for pheochromocytoma in patients with NF-1 and hypertensive symptoms to enable timely intervention and prevent severe complications.

## Introduction

Neurofibromatosis type 1 (NF1), also known as von Recklinghausen’s disease, is an autosomal dominant disorder that presents with multiple neurocutaneous manifestations, including café-au-lait spots, axillary or inguinal freckling, and neurofibromas [1-3]. This condition affects approximately one in 3,000 individuals worldwide and results from mutations in the NF1 gene on chromosome 17, which encodes neurofibromin - a critical tumor suppressor protein that regulates Ras signaling [4]. In the context of pheochromocytoma, tumor development is believed to result from biallelic inactivation of the NF1 gene in chromaffin cells, leading to unchecked Ras/MAPK pathway activation and cellular proliferation. In addition to its classic cutaneous and neurological features, NF1 can involve diverse organ systems, leading to skeletal dysplasias, optic pathway gliomas, and a heightened risk of both benign and malignant neoplasms [5,6].

Among the more uncommon yet clinically significant manifestations of NF1 is pheochromocytoma, a catecholamine-secreting neoplasm that arises from chromaffin cells within the adrenal medulla [7]. Notwithstanding, pheochromocytomas occur in an estimated 0.1-5.7% of NF1 patients; they carry the potential for severe cardiovascular events, which include hypertensive crises, arrhythmias, and cerebrovascular accidents if not identified and managed promptly [8-10]. The reported prevalence of pheochromocytoma in NF1 is wide due to variability in screening methods and diagnostic awareness across studies.

In this case report, a 50-year-old woman with NF1 is described who presented with a right adrenal mass subsequently diagnosed as pheochromocytoma. This presentation underscores the importance of early clinical suspicion and timely diagnostic workup in patients with NF1 who exhibit suggestive symptoms. Furthermore, it emphasizes the need for prompt therapeutic interventions to mitigate life-threatening complications. To our knowledge, there is limited literature from South Asia documenting large adrenal pheochromocytomas in patients with NF1 presenting with non-classical symptoms. By detailing this case-featuring a 10 cm adrenergic tumor in a patient with longstanding hypertension, diagnostic delay, and coexisting diabetes, we aim to contribute region-specific insight to this rare clinical association. In doing so, we also highlight the value of a multidisciplinary management strategy to optimize patient outcomes [11,12].

This article was previously presented as a poster at the International Endocrine Society 2024 on 23 November 2024.

## Case presentation

A 50-year-old woman presented with a one-year history of recurrent right flank pain, an unintentional weight loss of approximately 8 kg over the past three months, and multiple nodular skin lesions persisting for approximately three decades. The flank pain was insidious in onset and dull in nature, occasionally intensifying in severity and radiating across the entire abdomen. Although the pain was not clearly associated with food intake, it was partially relieved by bending forward or taking analgesics. She also experienced intermittent palpitations, sweating, and headaches, but denied chest pain, light-headedness, increased urinary frequency, or loss of consciousness during these episodes.

Upon further inquiry, the patient revealed that she had multiple, painless skin lesions defined as soft nodules on her face, neck, trunk, and extremities. She mentioned that these lesions first appeared in early adulthood and progressively increased in number, yet did not significantly interfere with her daily activities. Her family history indicated a hereditary component, as similar cutaneous lesions were reported in her father, siblings, and children.

Her medical history includes type 2 diabetes mellitus for two years, managed initially with oral hypoglycemics and currently with insulin, and hypertension for five years, controlled with a combination of amlodipine and olmesartan. She underwent a hysterectomy five years ago for a uterine fibroid.

**Figure 1:**
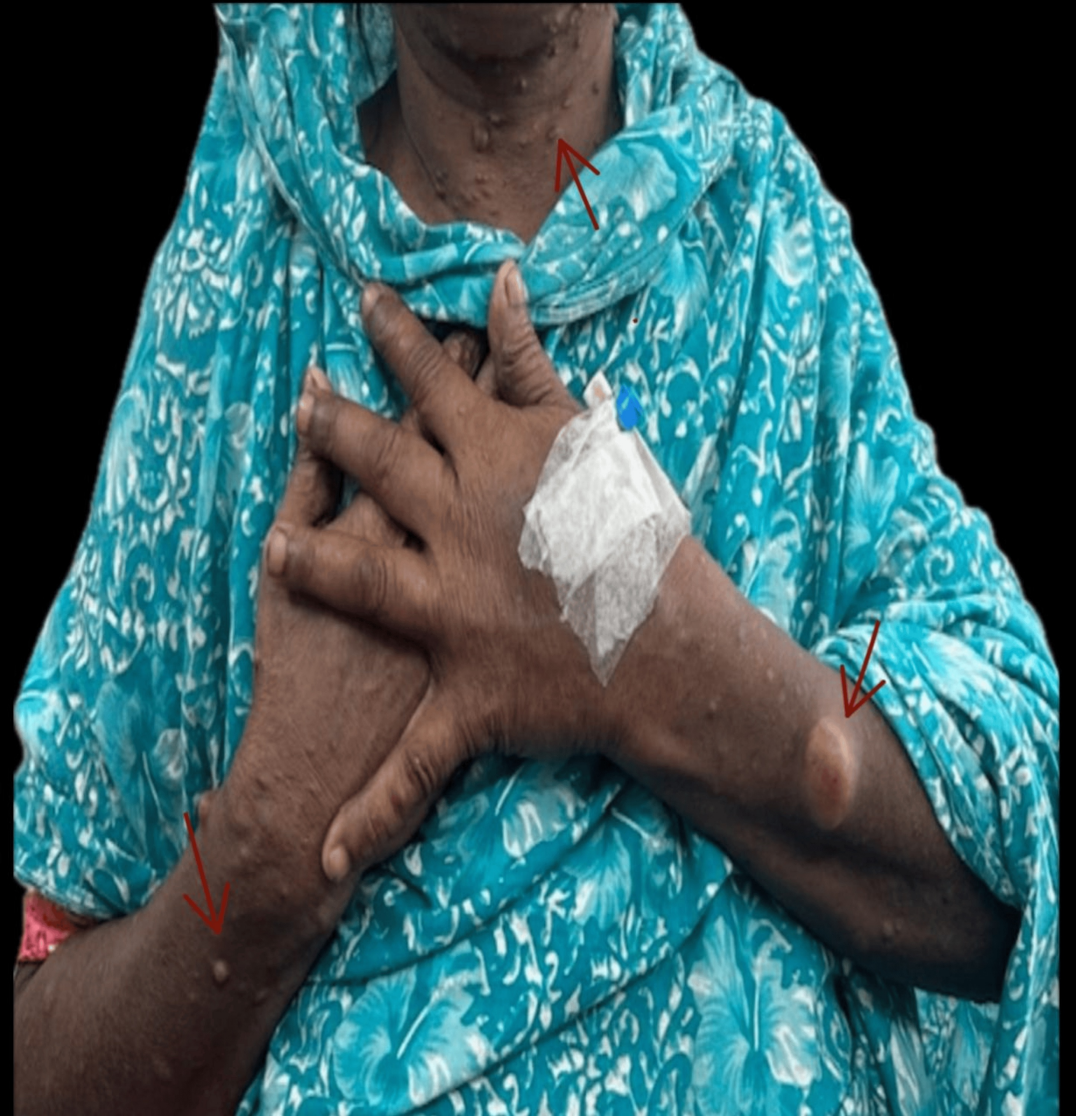
Multiple cutaneous neurofibromas

On physical examination, she appeared ill and anxious, with a below-average body habitus. Her blood pressure measured 150/100 mmHg, without any postural drop, and her pulse was regular at 110 beats per minute. Dermatological assessment revealed multiple well-circumscribed, soft, brown nodules scattered across her entire body, as shown in Figure 1, in addition to axillary and inguinal freckling. A bimanually palpable, ballotable, firm mass was identified in the right flank, aligning with the right kidney. Other systemic examinations, including cardiovascular, respiratory, and neurological examinations, were unremarkable. Ophthalmological assessment via slit-lamp revealed Lisch nodules, further supporting the diagnosis of NF1.

**Figure 2:**
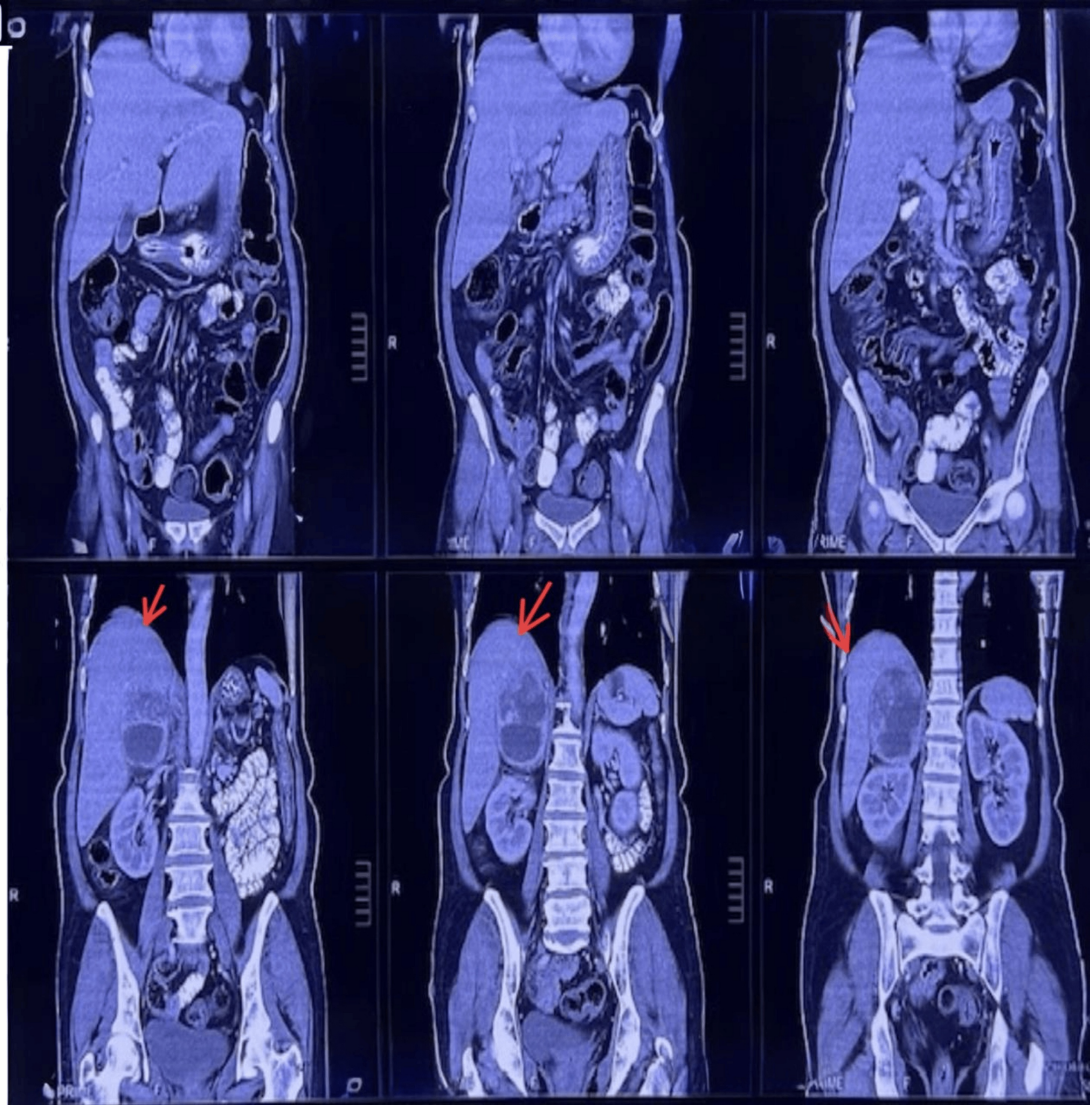
CT scan with adrenal protocol showing a right adrenal mass in a patient with neurofibromatosis type 1

Initial laboratory investigations showed elevated random blood glucose levels, while renal and hepatic function tests remained within normal limits. As shown in Table 1, the urinary metanephrine levels were significantly elevated. Prompted by the physical findings, an abdominal ultrasonography was performed, revealing a right adrenal mass. A contrast-enhanced CT scan with adrenal protocol confirmed a large, heterogeneously enhancing right adrenal lesion measuring 10 × 6 cm (Figure 2). The mass demonstrated a peak post-contrast enhancement of approximately 137 Hounsfield units (HU), supporting the diagnosis of pheochromocytoma. A 99mTc MIBI SPECT-CT scan confirmed the benign nature of the lesion, showing no abnormal radiotracer uptake. Differential diagnoses, such as adrenal adenoma, adrenocortical carcinoma, or metastatic lesions, were considered. However, the lesion's size, heterogeneous contrast enhancement, and biochemical profile - marked elevation of urinary metanephrines - strongly favored a diagnosis of pheochromocytoma.

**Table 1:** Patient laboratory test results RBS: red blood cell; WBC: white blood cell

Parameters	Patient values	Reference range (units)
Hemoglobin	13.4 gm/dL	12.1-15.1 gm/dL (females)
WBC	10,700/cumm	4,000-11,000/cumm
Platelets	358,000/cumm	150,000-450,000/cumm
RBS	13.2 mmol/L	<7.8 mmol/L
Serum Creatinine	1.01 mg/dL	0.6-1.2 mg/dL
Urinary Metanephrine	4230.5 microg/day	<900 microg/day

In view of the adrenal mass and catecholamine excess, multiple endocrine neoplasia type 2A (MEN2A) and von Hippel-Lindau (VHL) syndrome were also considered as differential genetic syndromes. However, there were no clinical or biochemical features suggestive of medullary thyroid carcinoma, primary hyperparathyroidism, or central nervous system (CNS) tumors. Thyroid examination, serum calcium levels, and imaging did not reveal additional findings to support MEN2A or VHL. The absence of relevant family history, endocrine abnormalities, or supporting radiologic features further reduced the likelihood of these syndromes.

Based on these findings, the patient was diagnosed with pheochromocytoma associated with NF1, alongside her existing type 2 diabetes mellitus and hypertension. Her management involved a multidisciplinary team comprising endocrinologists, radiologists, anesthesiologists, surgeons, and ophthalmologists, who collectively guided the diagnostic approach, preoperative optimization, and surgical planning. The patient was admitted for preoperative optimization, including alpha-adrenergic blockade using oral doxazosin 4 mg once daily, followed by propranolol 20 mg twice daily to control persistent tachycardia. She subsequently underwent elective open right adrenalectomy under general anesthesia without intraoperative complications. Postoperatively, her blood pressure normalized, and she was discharged on postoperative day seven. At three-month follow-up, she remains normotensive and well, under ongoing surveillance with endocrinology and surgical teams. This case underscores the rare but clinically significant association of NF1 with pheochromocytoma and highlights the significance of timely recognition and management to avert severe complications.

## Discussion

NF1 is an autosomal dominant disorder with an estimated incidence of one in 3,000 live births. It is characterized by cutaneous, ocular, and systemic manifestations, including neurofibromas, café-au-lait spots, axillary or inguinal freckling, and Lisch nodules of the iris. NF1 is caused by mutations in the NF1 gene located on chromosome 17, resulting in the loss of neurofibromin, a tumor suppressor protein that regulates the Ras signaling pathway [13].

Pheochromocytomas, rare catecholamine-secreting tumors, occur in 0.1%-5.7% of patients with NF1, significantly higher than in the general population [14]. These tumors can present with paroxysmal or sustained hypertension, headaches, palpitations, and sweating due to excess catecholamine secretion. If left untreated, pheochromocytomas can lead to life-threatening complications, such as hypertensive crises, arrhythmias, or stroke [14].

In the presented case, the patient presented with recurrent abdominal pain, weight loss, episodic palpitations, sweating, and headaches. Although these symptoms are nonspecific, the presence of a palpable right flank mass and multiple neurofibromas raised the suspicion of a pheochromocytoma associated with NF1. The patient’s dermatological findings of axillary freckling and nodular neurofibromas were consistent with NF1 [13,14]. However, diagnosis of pheochromocytoma in NF1 can be challenging due to symptom overlap with other NF1-related conditions, often resulting in delayed detection and management. Studies have noted that these tumors are frequently discovered incidentally or after significant diagnostic delays [8].

The diagnosis of pheochromocytoma was confirmed through imaging and functional studies. Contrast-enhanced CT of the abdomen revealed a large, heterogeneously enhancing right adrenal mass, consistent with pheochromocytoma. Pheochromocytomas typically enhance >110-130 HU on contrast CT. The 137 HU peak in our case further reinforced the diagnosis. Functional imaging with 99m Tc MIBI SPECT-CT demonstrated no increased radiotracer uptake, suggesting a benign etiology. Biochemical tests, such as plasma or urinary metanephrines, are crucial in the diagnosis of pheochromocytomas, as they have high sensitivity for detecting catecholamine excess [15,16].

The pathogenesis of pheochromocytoma in NF1 involves a two-hit mechanism: the inherited NF1 mutation, followed by somatic loss of the second NF1 allele in chromaffin cells. This results in dysregulated Ras signaling and increased proliferation of chromaffin cells, leading to tumor formation [15]. Unlike sporadic pheochromocytomas, NF1-associated pheochromocytomas are typically benign and less likely to be metastatic [13,15]. In NF1, pheochromocytomas generally belong to molecular cluster 2, characterized by adrenergic tumors with elevated metanephrine levels. This cluster is associated with mutations in kinase-signaling genes, including NF1, RET, and TMEM127, and typically exhibits a more differentiated and less metastatic behavior. Our patient’s elevated total metanephrines (4,230.5 µg/day) and NF1 background support this classification. While cluster 2 pheochromocytomas, such as those in NF1, are generally benign, tumor size remains a critical prognostic factor. Lesions >6 cm carry a higher risk of future metastasis, including late metastases occurring years after resection [17]. This reinforces the need for long-term follow-up in our patient.

Surgical resection is the definitive treatment for pheochromocytoma. However, preoperative management is crucial to prevent hypertensive crises during surgery. Alpha-adrenergic blockade, usually with phenoxybenzamine or doxazosin, is initiated to control blood pressure and prevent catecholamine surges. Beta-blockers may be added to manage tachycardia, but only after adequate alpha blockade to avoid unopposed alpha-adrenergic stimulation [16]. In this case, the patient was stabilized with alpha-blockade before undergoing planned adrenalectomy [13,14].

The prognosis for NF1-associated pheochromocytoma is generally favorable, with low recurrence rates following surgical resection. However, long-term follow-up is essential due to the risk of recurrence or development of other NF1-associated tumors, such as malignant peripheral nerve sheath tumors or gastrointestinal stromal tumors. Genetic counseling is also recommended for patients and their families to understand the hereditary nature of NF1 and its implications [13,16]. Although genetic testing for NF1 mutations was not performed due to financial and infrastructural limitations in our setting, the diagnosis was established clinically based on the National Institutes of Health (NIH) criteria. The patient also had a strong family history of similar cutaneous manifestations, further supporting a hereditary NF1 diagnosis.

Limitations of this report include the lack of genetic sequencing due to limited access and unavailability of standard functional imaging modalities, such as 123I-MIBG or DOTATATE PET-CT. Despite these constraints, the case provides important clinical and diagnostic insights for similar resource-limited contexts. To our knowledge, few cases from resource-limited settings have documented such a large pheochromocytoma in an NF1 patient, with emphasis on biochemical profiling, radiologic correlation, and multidisciplinary management despite limited resources.

## Conclusions

This case underscores the importance of maintaining a high index of suspicion for pheochromocytoma in patients with NF1, especially when presenting with subtle or chronic symptoms. Despite limited resources, timely diagnosis and coordinated multidisciplinary care enabled a successful outcome, highlighting the value of clinical vigilance and adaptable strategies in such settings.
